# Examining the Effects of Discrete Trials, Mass Trials, and Naturalistic Environment Training on Autistic Individuals Using Repeated Measures

**DOI:** 10.7759/cureus.53371

**Published:** 2024-02-01

**Authors:** Tami Peterson, Jessica Dodson, Alicia Hisey, Robert Sherwin, Frederick Strale

**Affiliations:** 1 Applied Behavior Analysis, The Oxford Center, Brighton, USA; 2 Physical Therapy, The Oxford Center, Brighton, USA; 3 Emergency Medicine, Wayne State University School of Medicine, Detroit, USA; 4 Biostatistics, The Oxford Center, Brighton, USA

**Keywords:** repeated-measures design, applied behavior analysis, naturalistic environment training, mass trials, discrete trial training, autism

## Abstract

Introduction: Behavioral interventions based on applied behavior analysis (ABA) form current evidence-based practices in treating autism spectrum disorder (ASD). Research is scarce relative to the broad effects of intensive repetitive, discrete trial training, and mass trials combined with a naturalistic environment as measured by overall general target behaviors. The primary objective of this study was to evaluate the effectiveness of a mixed behavioral model consisting of discrete trial training and mass trial interventions in the naturalistic environment, using a repeated measures design with a retrospective snapshot cohort of 93 individuals with autism.

Methods: A repeated measures analysis tracked 89 autistic children with four adult autistic individuals over seven time points during a three-month snapshot period from March 19, 2023, to June 11, 2023. This study determined the effectiveness of applied behavior analysis (ABA) interventions combining discrete trial training, mass trials, and naturalistic environment training on mastered broad target behaviors in autistic individuals using a mixed (between and within) ANOVA statistical design.

Results: Mixed (between and within) ANOVA indicated overall statistical significance, F (6,674)=45.447, p<0.001, partial eta squared=0.365 across time. These results indicated a large effect size. Multiple comparisons showed statistical significance (p<0.001) on all 21 multiple comparisons between timepoints. There was also a significant interaction effect with time × age category, F (24,474)=2.961, p<0.001, partial eta squared=0.130. These results also indicated a large effect size.

Conclusions: Autistic individuals who received applied behavior analysis combining discrete trial training, mass trials, and naturalistic environment training intervention demonstrated statistically significant improvement in target behaviors over the three-month snapshot period, the most prominent being in the 13-16 years age category.

## Introduction

According to the University of California Davis Medical Center, treating individuals with autism spectrum disorder (ASD) will cost the United States approximately $500 billion (about $1,500 per person) by 2025. [1]. The scientific tenets of applied behavior analysis (ABA) point toward ongoing evaluation of these interventions in unique settings and the individual recipient's needs [2]. Estimates show that ABA treatments, such as early intensive behavioral interventions (EIBI), may save hundreds of thousands of dollars in public funds for individuals with ASD over the period that they are eligible for services under the Individuals with Disabilities Education Act (IDEA) (ages 3-22 years). Behavioral interventions based on ABA form the most current evidence-based practices in treating (ASD) [2]. Experimental treatments may be applied in educational, community, and clinical settings to develop functional skills that afford as much independence as possible for individuals with ASD to live fulfilling lives [2].

Over 30 years of accumulated research suggests that applied behavior analysis ABA interventions are at the forefront of evidence-based therapy supporting the development of individuals with ASD [3]. Despite a plethora of evidence for the effectiveness of ABA, consumer concerns and misconceptions persist [4]. The National Professional Development Center on Autism Spectrum Disorder (NPDC), the National Autism Center (NAC), and various current articles recommend that consumers of ABA educate themselves on the “practice” of ABA and what constitutes effective service delivery of evidence-based interventions [4].

For many reasons, reporting of general ABA broad effectiveness with large N designs using a mixed behavioral model, e.g., discrete trial training, mass trials, and a naturalistic environment, is needed. It can continuously inform families, educators, clinicians, and policymakers about the benefits and limitations of ABA with autistic individuals [5]. It can also provide evidence-based support for using (ABA) interventions as medically necessary and reimbursable. Given the abundance of small N studies delineating the positive effects of (ABA) therapy, extensive large N studies of general ABA broad effectiveness can lead to further research to improve quality and outcomes. In addition, there is a lack of published studies using repeated measures designs.

The primary objective of this study was to extend the research to evaluate the effectiveness of a mixed behavioral model using a repeated measures design with a retrospective snapshot cohort of 93 autistic individuals treated with discrete trial training and mass trials in a naturalistic environment over seven time points covering three months [6]. It is hypothesized that the child cohorts treated with the mixed model, consisting of discrete trial training and mass trial interventions in the naturalistic environment, will demonstrate statistically significant progress toward general target behavioral goals over time.

## Materials and methods

Study setting and participants

General target mastery data were collected daily by a team of multiple (three to five) behavioral technicians per child for 100 total individuals with autism using a large N design via retrospective chart reviews contained within the “catalyst” tracking software [5]. Behavior analysts administered a mixed model of discrete trial training, mass trials, and naturalistic environment treatment for three months between March 19, 2023, and June 11, 2023. General target mastery data were collected for 89 children and four adults, with seven missing values.

Inclusion and exclusion criteria

All male and female participants, any autistic individual between the ages of one and 73 years, medically cleared for treatment, official diagnosis of autism spectrum disorder by a psychiatrist, psychologist, or primary care physician were included in this study.

The individuals who do not have a diagnosis of ASD, individuals who have a medical condition or disability that makes ABA therapy unsafe, have a history of abuse, neglect, or trauma that may interfere with their ability to benefit from ABA therapy, individuals who received another intervention that is incompatible with ABA therapy, and the family and the provider who cannot resolve important issues related to the treatment plan were excluded from this study.

Data collection

Catalyst is a commercial electronic data collection tool that assists interventionists with capturing and analyzing copious quantities of behavioral data in a way that replicates how behavior technicians collect conventional paper data collection. Board-certified behavior analysts (BCBAs) created a treatment plan for each child and implemented programs and data collection methods for behavior reduction and skill acquisition [7].

Behavior technicians assigned to specific autistic individuals used real-time data-stamping procedures to enter data the second the behavior was observed. The behavior technician created an operational definition for the problem behavior and selected continuous (frequency, duration) measurement systems using a portable electronic device (an iPad; Cupertino, CA: Apple Inc.). Researchers then had access to those data online for analysis and reporting.

All autistic individuals were seen and treated at The Oxford Centers (TOCs) in Brighton and Troy, MI. TOCs are specialized in the mixed methods approach to applied behavior analysis (ABA), employing discrete trial training, mass trials, and naturalistic environment training treatment modalities. Before training, each individual received a treatment plan developed by one of eight BCBAs based on the individual’s needs and goals. The individual was assigned to one of 83 behavioral technicians and had a three to five behavioral technician team over the three months. Appropriate materials were selected and set in rooms where individual discrete trial training and mass trials occurred or in a naturalistic setting where the participant interacted with others and experienced functional and meaningful real-world situations. Each behavioral technician was assigned to a different participant daily, receiving, on average, four to seven hours of treatment per day for a minimum of 25 hours a week.

Behavioral technician teams collected specific behavioral and skill data related to antecedents, behavior, and consequences of behavior. They observed progress, noted the fading of prompts and reinforcements as the participant attempted to master the skill, and assessed whether the participant was generalizing and maintaining the skill. Data were entered into a handheld “catalyst” database and aggregated and updated daily into a central database.

Outcome measures

The dependent variable was the multiple rater composite scores for the number of aggregated general target behaviors mastered measured at seven time points every two weeks over the three months. In a broad sense, these “general aggregate target behaviors,” as defined by BCBAs and behavioral technicians at the Oxford Center, involved daily living skills, including daily repertoires, organization, time management, eating-related skills, toileting, and hygiene routines. Participants learned expressive communication skills, which involved speaking with words and phrases, expanding vocalizations to using more complex vocabulary, improving conversational skills, greeting people, responding to greetings, asking for assistance, and requesting things. Receptive language skills were also emphasized, such as following directions and identifying stimuli upon request.

Social skills were trained, including taking turns playing with friends, sharing, displaying assertiveness, interacting with peers, and responding appropriately to new people. Community skills in naturalistic environments involve responding to a cashier in a store, purchasing items, money management, shopping for groceries, ordering food in a restaurant, speaking to a policeman, safe walking on a sidewalk, safe playing at a park, and safety skills with strangers.

The independent variable was time, with seven levels (Time 1 {baseline}, Time 2 {after two weeks}, Time 3 {after four weeks}, Time 4 {after six weeks}, Time 5 {after eight weeks}, Time 6 {after 10 weeks}, and Time 7 {after 12 weeks}). Given that each participant’s treatment plan varied, in a general sense, the mixed model treatment administered consisted of discrete trial training combined with massed trial instruction and a naturalistic environment treatment, with reinforcers chosen for strength, clear contingencies, and repetition to teach new behaviors.

Luiselli noted that naturalistic teaching promotes the generalization of skills to everyday settings where those skills are required, thus enhancing the generalization of language, social, and play skills [8]. Compared with more structured approaches, naturalistic teaching better generalizes critical skills to the natural setting. These procedures happen within the context of everyday activities, making learning more fun and enhancing the individual's willingness to engage in learning.

This instills confidence that these procedures are a viable, evidence-based method in providing therapy to autistic individuals. ABA interventionists teach responses, creating contact with natural reinforcers, allowing the individual’s interests to direct and pace teaching. Naturalistic environments also embed education within everyday activities, incorporating prompts to be transported to new situations. Some skills can be learned in a controlled setting before transitioning to a naturalistic setting [8].

This retrospective-repeated measures design used a one-group pretest-posttest design, which will assess the clinical application of ABA with functional analysis and discrete trial training in a naturalistic setting to increase the occurrence of mastered target behaviors and decrease problematic behaviors with a three-month snapshot (March 19, 2023 through June 11, 2023) sample [9]. Repeated measures deal with outcomes measured on the same experimental unit at different times or under other conditions, with each participant serving as their control [10,11].

Sample size determination

A retrospective power analysis was conducted using GPower 3*1 (Düsseldorf, Germany: Faul et al., Heinrich Heine University Düsseldorf) and indicated n=14 participants would be required to demonstrate a high group effect size (0.80) with an alpha (α)=0.05 using a mixed (between and within) ANOVA, with a power equal to 0.9938. Given these parameters, an acceptable sample size criterion is highly likely [12].

Statistical methods 

SPSS version 29.0 (Armonk, NY: IBM Corp.) was used for all descriptive and inferential statistics [13]. Alpha (α) was set at 0.05. If p-values were less than 0.05, the null hypothesis was rejected, implying statistical significance. Demographics and baseline characteristics were summarized. Summary statistics for the categorical variables gender, race/ethnicity, and the continuous variables age, Time 1, Time 2, Time 3, Time 4, Time 5, Time 6, and Time 7 (mean and standard deviation, median, range, and skew) were generated.

A mixed (between and within) ANOVA was used to determine the overall statistical significance between the (Time 1 to Time 7) measurements, as well as any interaction effects. If an overall significant omnibus F statistic was detected (p<0.05) within the mixed (between and within) ANOVA, a step-down analysis was performed using resampling multiple comparison procedures in the form of bootstrapped paired tests (1000 replications). Using bootstrapping with paired t-tests, resampling methods mitigate potential multiplicity, thereby reducing familywise error rate (FEW) likelihoods [14].

The Bonferroni correction was also used as α=0.05/21=0.0024. Therefore, with these multiple comparisons, if p<0.0024, a null hypothesis was rejected, and statistical significance was inferred [14]. If an overall significant omnibus interaction F statistic is detected (p<0.05) within the mixed (between and within) ANOVA, a step-down analysis will be performed using interaction contrasts comparing the between subjects’ factor with the within subjects’ factor to determine precisely where the significant differences (effects) came about [10]. Effect sizes in the form of Cohen's d were also reported, and threats to internal validity were noted [15-17].

Each valid score (n=93) in the dataset was an equally weighted composite score of the number of aggregated general target behaviors mastered, measured at seven time points, which were the average of the multiple (three to five behavioral technician) ratings. Interclass correlations (ICC) were used to measure the degree of agreement between the multiple raters, contributing to the seven timepoint composite variables.

Interobserver reliability

A two-way random effects model was computed where people's effects and measures effects are also random. We used the ICC two-way random effects model (2), which is used when multiple measurements are made from each averaged rater. The ICC (2) value was 0.860 (95% CI: 0.758-0.915), indicating excellent agreement between the raters. This value was more significant than the average Pearson r (0.750), suggesting that the ICC (2) was more sensitive to the variability among raters and measurements. Cronbach’s alpha for the seven time point variables was r=0.91 indicating a high internal consistency reliability.

Ethical approval

This research study was conducted retrospectively from data obtained via chart review for clinical purposes. The study was submitted to the Western Copernicus Group-Institutional Review Board (WCG-IRB) for review and received an exemption (#1-1703366-1). The authors hereby certify that the analysis was performed in accordance with the ethical standards as put forth in the 1964 Declaration of Helsinki and its later amendments or comparable ethical standards. Please note that since obtaining the ClinicalTrials.gov Identifier: NCT06043284, Oxford Recovery Center (ORC) has changed its name to The Oxford Center (TOC) (other study ID numbers: OxRS-01-2021).

## Results

For the sample of 100 autistic children (M=8.8817, SD=8.05), the median was 7, the minimum was 1, and the maximum was 73. There were seven missing values, and 74 males (74.0%) and 25 females (25.0%), with one missing value. There were 75 Whites (75.0%), four Asians (4.0%), three Hispanics (4.0%), 12 Middle Eastern (12.0%), and six African Americans (6.0%), with no missing values. In terms of age categories, 18 (18.0%) were in the one to four-year category, 39 (39.0%) were in the five to eight-year category, 20 (20.0%) were in the nine to 12-year category, 12 (12.0%) were in the 13 to 16-year category, and four (4.0%) were in the 17 to 73-year category. Four subjects were over 17 years old, i.e., 18 years, 20 years, 25 years, and 73 years. There were seven missing values.

Descriptive statistics for Time 1 (baseline), Time 2 (after two weeks), Time 3 (after four weeks), Time 4 (after six weeks), Time 5 (after eight weeks), Time 6 (after 10 weeks), and Time 7 (after 12 weeks) measurements are presented in Table 1. Please note that mean and median increase from Time 1 to Time 7.

**Table 1:** Descriptive statistics for repeated measurements. n=total number

Variables		Targets mastered Time 1-baseline	Targets mastered Time 2-2 weeks	Targets mastered Time 3-4 weeks	Targets mastered Time 4-6 weeks	Targets mastered Time 5-8 weeks	Targets mastered Time 9-11 weeks	Targets mastered-Time 7-12 weeks
n	Valid	93	91	91	91	90	92	92
	Missing	7	9	9	9	10	8	8
Mean		1.3226	3.9670	7.7143	13.3874	17.5250	21.1593	27.1332
Median		0.0000	2.0000	4.0000	8.0000	11.0000	12.0000	18.0000
Standard deviation		2.56401	4.88581	9.64657	16.35497	19.60423	22.42591	26.56894
Skewness		2.560	1.444	1.809	1.840	1.654	1.360	1.451
Standard error of skewness		0.250	0.253	0.253	0.253	0.254	0.253	0.251
Minimum		0.00	0.00	0.00	0.00	0.00	0.00	0.00
Maximum		13.00	19.00	47.00	70.00	90.00	102.00	128.00

Mixed ANOVA - main effects

There was a significant main effect (sphericity assumed) on the dependent variable (general targets mastered) across time, (F{6,474} = 45.447, p<0.001, partial eta squared=0.365), indicating an overall statistically significant effect (increase in general targets mastered) across the seven timepoints (levels) of the independent variable (time). The partial eta squared of 0.365 also indicates a large effect size.

Post hoc analyses

Post hoc analysis was conducted using a bootstrapped (1000 replications) paired t-test for multiple comparisons with a Bonferroni correlation (0.05/21) α=0024. The 21 multiple comparisons are presented in Table 2.

**Table 2:** Post hoc analysis with bootstrapped paired t-tests and effect sizes using Cohen's d.

Timepoint	Mean difference	Standard error (SE)	t-test	df	p-Value (two-tailed)	95% CI for mean difference	Cohen’s d
1 vs 2	-2.644	0.375	-7.05	89	<0.001	-3.48, -1.94	-0.743
1 vs 3	-6.334	0.875	-7.24	89	<0.001	-8.27, -4.78	-0.763
1 vs 4	-12.025	1.602	-7.51	89	<0.001	-15.22, -9.26	-0.791
1 vs 5	-16.158	1.937	-8.34	89	<0.001	-19.97, -12.82	-0.879
1 vs 6	-19.606	2.214	-8.75	89	<0.001	-23.89, -15.70	-0.992
1 vs 7	-25.287	2.693	-9.60	89	<0.001	-31.13, -21.09	-1.012
2 vs 3	-3.689	0.692	-5.33	89	<0.001	-5.08, -2.41	-0.562
2 vs 4	-9.381	1.452	-6.50	89	<0.001	-12.26, -6.93	-0.681
2 vs 5	-13.514	1.772	-7.63	89	<0.001	-16.87, -10.57	-0.804
2 vs 6	-16.961	2.071	-8.19	89	<0.001	-20.76, -13.44	-0.863
2 vs 7	-23.203	2.513	-9.23	89	<0.001	-28.05, -18.78	-0.973
3 vs 4	-5.692	0.990	-5.75	89	<0.001	-7.80, -3.98	-0.606
3 vs 5	-9.825	1.296	-7.58	89	<0.001	-12.47, -7.53	-0.799
3 vs 6	-13.272	1.627	-8.16	89	<0.001	-16.26, -10.29	-0.860
3 vs 7	-19.514	2.108	-9.26	89	<0.001	-23.63, -15.54	-0.976
4 vs 5	-4.133	0.563	-7.34	89	<0.001	-5.22, -3.21	-0.774
4 vs 6	-7.581	0.975	-7.78	89	<0.001	-9.38, -5.79	-0.820
4 vs 7	-13.822	1.526	-9.06	89	<0.001	-16.95, -11.00	-0.955
5 vs 6	-3.448	0.624	-5.52	89	<0.001	-4.66, -2.34	-0.582
5 vs 7	-9.689	1.253	-7.73	89	<0.001	-12.48, -7.46	-0.815
6 vs 7	-6.242	0.928	-6.63	89	<0.001	-8.28, -4.60	-0.709

Results indicated that for all 21 comparisons across the independent variable (time) dimension, all p-values were <0.05 and were statistically significant at α=0.05. All effect sizes, as reported by Cohen’s d, were >0.550, indicating medium to large effect sizes.

Mixed ANOVA - interaction effects (time × age)

There was a significant interaction effect (sphericity assumed) on the dependent variable (general targets mastered) across the time and age category, F(24,474)=2.961, p<0.001, partial eta squared=0.130, indicating a statistically significant interaction effect detected across the seven timepoints of the independent variable (time) with age category. Statistically significant effects were detected in the toddlers (one to four years) and adolescent (13-16 years) age groups. The partial eta squared of 0.130 indicates a large effect size.

## Discussion

Discrete trial training is an applied behavior analytic modality that simplifies complexity by taking large, gross tasks, reducing them to small, individualized tasks, and teaching them with straightforward and systematic methods. Mass trials are a method within discrete trial training that includes repeatedly presenting the same stimulus until the learner responds correctly. Naturalistic environment training is a form of ABA that teaches behavioral skills within the natural context of a learning environment. The respective learner’s individual preferences and partialness serve as the motivation. The effects of a blend of discrete trial training, mass trials, and naturalistic environment training in autistic children are noteworthy as they can assist with various aspects of learner cognitive, language, social, and adaptive skills development. The benefits of discrete trial training include helping autistic children learn appropriate responses to different situations, which can enhance communication, their relationships with family, classmates, and peers, and overall quality of life. Acquisition of skills such as matching, discrimination, and imitation using this form of ABA can enhance learning that is difficult to acquire in naturalistic settings [18-25].

Mass trials assist autistic children with acquiring new behaviors more quickly and efficiently as exposure to the same or similar stimulus increases. This ABA method can help increase and retain learned behaviors over time by strengthening memory and improving recall abilities. Naturalistic environment training (NET) assists autistic children with generalization skills transferred from discrete trial training to different contexts, including people, materials, and settings. NET also helps with increased motivation, spontaneity, and engagement by utilizing reinforcements that occur naturally and are aligned with learner interests [18-25].

Given the general steady increase in our study with general target mastery over the designated three-month time points, precisely, the prevalence of effects during the toddler (one to four years) and adolescent (13-16 years) period, discovered with the interaction effect, an exploratory hypothesis may be put forth for future research. Inquiry into the different ages of participants is also needed [5]. Despite this, the statistically significant effects found in toddler (one to four years) and adolescent (13-16 years) age categories may be a chance occurrence and the result of a biased situation inherent with convenience samples [26-30]. This interaction effect needs to be interpreted cautiously as it may result from a type I error.

Ongoing studies of general ABA broad effectiveness, namely, with discrete trial training, mass trials, and naturalistic environment training, with large N studies, can lead to further research to improve quality and service. This study served to address consumer concerns and misconceptions and inform consumers on the “practice” of ABA and the effective service delivery of evidence-based therapies as requested by the National Professional Development Center on Autism Spectrum Disorder (NPDC) [31].

This research is consistent with the National Professional Development Center on Autism Spectrum Disorder (NPDC), the National Autism Center (NAC), and various current articles advising consumers of ABA to become well-versed on the “practice” of ABA and the characteristics of effective service delivery of evidence-based behavior analytic interventions. These recommendations can continuously inform families, educators, clinicians, and policymakers about the benefits and limitations of ABA with autistic children. Research must continue to support evidence-based practices and continued improvement [5].

Limitations

Although the findings of this research are informative, it is essential to point out its limitations. A non-random sample was used for this study; therefore, there is no ability to generalize beyond this sample. Due to data constraints, there is no delineation of possible statistically significant differences between the groups relative to discrete trial training, mass trials, and naturalistic environment training.

Seven threats to internal validity apply in pre-experimental research designs of this type. History points toward extraneous variables not part of the study or any external events that may affect outcomes. Maturation involves age-related bodily changes and includes age-related physical changes that can occur with time, such as hunger, tiredness, fatigue, wound healing, surgery recovery, disease progression, etc. Testing relates to the notion that the test may affect the children’s responses when tested again. These are less of an issue when the tests are routine. Instrumentation refers to any change in measurement ability, including any judge, rater, etc. Statistical regression is the tendency for individuals who score extremely high or low on a measure to score closer to the mean of that variable the next time they are measured on it. Selection refers to the potential bias in selecting participants who will serve in the experimental and control groups. Mortality refers to the differential loss of study participants, drop-out rate, or attrition [17]. This is a within-subjects design, whereby each subject serves as its control; therefore, there was no control group utilized for ethical purposes.

Also, this is a snapshot study that covers three months, and it will be informative to assess these research subjects over a longer time longitudinally. This study uses retrospective data, and while the single group pre-post design is pre-experimental, a prospective study is warranted for future investigation. Ethical issues preclude utilizing a control group (no treatment) for autistic children. Also, there appears to be a need in the literature concerning the analysis of discrete trial training and naturalistic environment training with repeated measures that call for future inquiry.

## Conclusions

The primary objective of this study was to evaluate a mixed applied behavior analytic model's effectiveness, combining discrete trial training, mass trials, and a naturalistic environment on the number of aggregated general target behaviors mastered in autistic individuals using a repeated measures analysis. This is the first piece of research utilizing a mixed model of discrete trial training, mass trials, and naturalistic environment treatment with a measured effect on general target mastery information using a large N design with repeated measures. The statistical results showed that these interventions significantly increased general aggregate target behaviors over seven time points. We observed statistically significant increases in mean and median measurements of the number of multiple raters composite general target behaviors achieved per session. The multiple comparisons between time points indicated an upward trend of improvement and statistically significant differences between time points with medium to large effect sizes, the most prominent being in 13-16 years age category.
